# Avoidance behaviour toxicity tests should account for animal gregariousness: a case study on the terrestrial isopod *Porcellioscaber*

**DOI:** 10.3897/zookeys.1101.76711

**Published:** 2022-05-18

**Authors:** Primož Zidar, Žiga Fišer

**Affiliations:** 1 Department of Biology, Biotechnical Faculty, University of Ljubljana, Večna pot 111, SI-1000 Ljubljana, Slovenia University of Ljubljana Ljubljana Slovenia

**Keywords:** Aggregation, ecotoxicology, locomotion, Isopoda, Oniscidea, woodlice

## Abstract

Avoidance behaviour enables woodlice to escape suboptimal environmental conditions and to mitigate harmful effects of pollutants. However, several studies have shown that at least in some woodlice species the tendency to aggregate can lead to suboptimal responses as the between-conspecific attraction can outweigh the aversive stimuli. The present study evaluated the influence of gregariousness on the behaviour of *Porcellioscaber* in a heterogeneously polluted environment. The hypothesis was that the tendency for aggregation outweighs the tendency for exploratory activity, therefore animals in groups will be less active. Consequently, this will affect their avoidance of polluted environmental patches. To test this hypothesis, isolated individuals or pairs of individuals were monitored in free-choice arenas where animals could select between uncontaminated and pyrethrin-contaminated soils. Animals were video recorded for 3 h in darkness using infrared light and analysed for avoidance behaviour and locomotor activity. In general, isolated animals were more locomotory active and avoided the contaminated soil more than paired animals. It can be concluded that aggregation behaviour suppresses exploratory behaviour and consequently also the avoidance of polluted environments. This should be accounted for when interpreting results of avoidance tests with groups of gregarious animals, which may underestimate the effect of pollutants.

## Introduction

In the modern world not only heterogeneous distribution of resources but also environmental pollution is forcing animals to an exploratory behaviour. The shorter the time animals spend in a polluted environment, the more likely are they to survive. To do so, sensing, locomotor activity, spatial orientation, and appropriate storage of information about the new environment are crucial.

There is no doubt that terrestrial isopods can sense certain pollutants as many studies have shown their avoidance of soil or food polluted with metals (Zidar et al. 2003, 2005; Loureiro et al. 2005), veterinary pharmaceuticals (Žižek and Zidar 2013), pesticides (Loureiro et al. 2005; Santos et al. 2010; Zidar et al. 2012), char (Madžarić et al. 2018), nanoparticles (Zidar et al. 2019), or a mixture of pollutants (Loureiro et al. 2009). Terrestrial isopods have simple eyes that can perceive only light/dark contrasts, so these animals rather rely on their two pairs of chemosensory antennae for spatial orientation tasks such as finding food and suitable microhabitats, as well as to communicate with conspecifics and avoid predators (Schmalfuss 1998; Thiel 2010). As ancestrally aquatic animals, woodlice evolved many adaptations enabling their life in terrestrial habitats (Schmalfuss 1998; Hornung 2011; Richardson and Araujo 2015; Sfenthourakis et al. 2020). Most species are either endogean or epigean and nocturnal (Schmalfuss 1998). The latter are active during the night-time and spend the day sheltering in dark and damp places to avoid desiccation (Hassall and Tuck 2007; Tuf and Jerabkova 2008; Hornung 2011). Another behavioural adaptation to the terrestrial environment is gregariousness, i.e., a tendency to aggregate or associate with conspecifics (Hornung 2011; Broly et al. 2013). This behaviour significantly reduces water loss (Allee 1926; Broly et al. 2014), promotes body growth (Takeda 1984), increases reproductive performance (Souty-Grosset et al. 1990), and protects against predators (Broly et al. 2013). However, several studies have shown that at least in some woodlice species the tendency to aggregate can lead to suboptimal outcomes (Devigne et al. 2011; Broly et al. 2012, 2015). This is because the attraction between conspecifics can outweigh individual preferences to other environmental stimuli. Loureiro et al. (2005) noticed that aggregation is also likely to affect woodlice avoidance to a polluted environment. The same was noticed by Zidar et al. (2012) in the study of woodlice behavioural response to insecticide pyrethrin.

Avoidance behaviour as an endpoint is frequently used in ecotoxicological studies to determine soil quality (ISO 2008, 2011; van Gestel et al. 2018). Avoidance tests are multiple choice experiments where a group of ten animals, usually earthworms, springtails, or isopods might select between two or more differently contaminated soils. The measured outcome of such tests is the number of animals on uncontaminated and contaminated soil after 48 h exposure. Advantages of this kind of avoidance tests are simplicity, short duration, and sensitivity that is comparable to acute or reproduction tests (Hund-Rinke and Wiechering 2001; Loureiro et al. 2005; Žižek and Zidar 2013; Madžarić et al. 2018; van Gestel et al. 2018). However, in gregarious animals like isopods, aggregation behaviour might seriously affect the results of avoidance tests.

In this study we evaluated the influence of gregariousness on the behavioural response of individuals in a heterogeneously polluted environment. We hypothesized that the tendency for aggregation outweighs the tendency for exploratory activity, therefore animals in a group will be less active. Consequently, this will affect their avoidance of contaminated soil. To test this hypothesis, we monitored the locomotor activity and avoidance response of the terrestrial isopod *Porcellioscaber* Latreille, 1804 in a heterogeneously polluted environment.

## Materials and methods

### Experimental animals

*Porcellioscaber* is one of the most frequently used species in toxicity testing (van Gestel et al. 2018). The parent population of animals used in this study originated from an unpolluted environment near the city of Kamnik (46°12'01.8"N, 14°35'31.7"E) in Slovenia (Europe). Animals were bred for several years in large glass containers with a mixture of limestone sand and soil at the bottom, at room temperature (20–23 °C), high relative air humidity (~ 95%) and a natural diurnal light regime. They were left to feed ad libitum on maple (*Acer* sp.) leaf litter, with regular additions of carrots and potatoes.

For the experiments, laboratory raised individuals were used. On the day of the recording, adult male specimens in the intermoult phase (Zidar et al. 1998) were selected from the breeding containers. Body size (measured from the anterior edge of the head to the base of the uropods) of the selected isopods ranged between 10 and 12 mm.

### Experimental set-up

**Figure 1.**
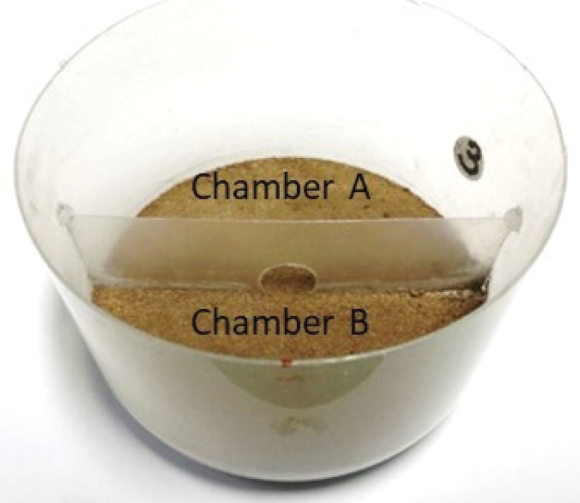
The two-chambered test arena for video tracking experiments with *Porcellioscaber*. Animals could select between uncontaminated (chamber **A**) and pyrethrin-contaminated soils (chamber **B**).

Isolated individuals or individuals accompanied by a conspecific were monitored in free-choice experiments where animals could select between uncontaminated and pyrethrin-contaminated soils. For this purpose, circular transparent polypropylene (PP) pots meant for food packaging (diameter 9.5 cm, height 6.0 cm) were used as test arenas. Arenas were divided into two equally sized chambers with a 3.5 cm high PP barrier (Fig. 1). In the middle of the barrier was a passage (diameter of 1.2 cm), which was large enough for the isopods to easily pass through. At the bottom of the arena there was a 10 mm thick plaster of Paris darkened with charcoal (Sørensen et al. 1997).

Approximately 20 min prior recording a 3.0 g of Lufa 2.2 soil (Speyer, Germany) was added on top of the plaster that was previously saturated with tap water. Soil was previously dried, grinded, and sifted through a 0.5 mm sieve. Homogeneously granulated substrate prevented additional tactile stimuli that can affect animals’ activity (Anselme 2013). In control groups uncontaminated soil was added to both test arena chambers simultaneously. In treatment groups uncontaminated and contaminated soils were added separately to each chamber in the following manner. First, the passage between the chambers was closed with an adhesive tape. Next, uncontaminated soil was added to one of the chambers (chamber A), previously marked with a number (Fig. 1). Afterwards, contaminated soil was added to the adjacent chamber (chamber B) and, finally, the adhesive tape blocking the passage was removed.

For each new recording a new substrate was prepared, both plaster and soil, but pots were re-used after thorough washing with tap water. This prevented any other chemical stimuli besides insecticides to affect animals’ activity. For example, it is known that pheromones in woodlice faeces promote aggregation behaviour (Takeda 1980).

### Soil contamination

Pyrethrins were used as a soil contaminant in all experiments in this study. Pyrethrins are chemicals of natural origin with insecticidal action that have been used to control pests indoor and outdoor since the early 1800’s (Casida 1980; Tod et al. 2003). They prolong the openness of sodium channels in nerve cells, which paralyses the animal at higher doses. Therefore, longer exposure to an insecticide reduces animal’s chances of survival.

Soil was contaminated for 1 h prior the recording with the insecticide product Flora Kenyatox Verde Plus (Unichem, Slovenia) which contained 0.2% of pyrethrin. The insecticide product was well shaken and 50 µL, 100 µL, 150 µL, and 200 µL was added to 20 g of dry soil and mixed well with a spatula. The obtained concentrations were 2.5, 5.0, 7.5, and 10.0 mL of insecticide formulation per kg dry soil which roughly correspond to 5.2, 10.4, 15.7, and 20.8 mg of pyrethrin per kg dry soil.

### Video recording and observation of animals

One-hour prior video recording animals were marked dorsally across several tergites with partly dried enamel white paint to increase the visual contrast between woodlice and the substrate. In the case of two animals per arena only one animal was marked. It was reported previously that some external markers (e.g., nail polish) may affect diurnal activity and food consumption in some isopod species (Drahokoupilová and Tuf 2012; Kenne et al. 2019). However, our previous experiments have shown that the marking we used is less persistent and has no effect on animal activity. Until recordings, animals were kept on moist plaster to prevent desiccation.

At the start of recordings one or two animals were placed in test arenas, always in chamber A containing uncontaminated soil. The animals were then continuously recorded for 3 h. In similar studies, animals’ activity was tracked for 2 or 4 hours (Bayley et al. 1997; Sørensen et al. 1997; Engenheiro et al. 2005). Our preliminary observations have shown that *P.scaber* is highly active up to 3 h after introducing into the arena but then its activity decreases due to habituation (Anselme 2013), therefore recording longer than 3 h is unnecessary.

To provide a recording environment isolated from the outside light and other unwanted perturbances, a specially designed recording box was used. The box measured 55, 100, and 100 cm in depth, width, and height, respectively. Animals were recorded only under infrared light (850 nm) to avoid any light-induced behaviour (Devigne et al. 2011; Broly et al. 2015). Videos were captured with two webcams (Logitech C920) simultaneously. Cameras have been modified to improve video quality for recordings in infrared light. Each camera recorded two arenas. A 140-led IR illuminator was used to ensure adequate illumination of test arenas.

Videos were captured in VirtualDub 1.10.4 at 5 frames per second and a Full HD resolution (1920 × 1080 pixels). One pixel corresponded to 0.13 mm.

### Video analysis

Videos were first analysed via video-tracking in Bonsai 2.4.0 (Lopes et al. 2015). The area of each test arena chamber was isolated by cropping. The white spot at the back of the marked animal was extracted from the background by thresholding and the spot’s centroid coordinates inside each chamber were determined for each video frame. In the case of paired animals only the marked animal was analysed. Altogether 44 isolated and 44 paired animals were analysed (*n* = 12 for 0 mg of pyrethrin per kg dry soil; *n* = 8 for 2.5, 5.0, 7.5, and 10.0 mg of pyrethrin per kg dry soil).

Next, videos were examined also manually. In this way a sequence of several hundred frames when animals were at rest with antennae close to the body and no detectable movements was selected. Based on allocations of the centroid between two consecutive frames in this sequence the upper limit for noise was determined (0.2 pixels or 0.022 mm per frame). The upper limit for non-locomotor activity (0.8 pixels or 0.088 mm per frame) was determined based on a sequence of several hundred frames when animals were feeding or digging. All larger allocations of the centroid between two consecutive frames (> 0.8 pixels) were considered as locomotor activity. Additionally, the number of visits to contaminated soil (chamber B) was counted.

Finally, raw trajectories were imported to MS Excel and used to calculate behavioural variables: the proportion of time that isopods spent on the uncontaminated soil (chamber A), the overall duration of locomotor and non-locomotor activity. Change in animal’s position during locomotor activity was calculated as path length. Average speed was calculated as the total path length divided by the total time of locomotor activity.

### Data analysis

All statistical analyses were performed in R 4.1.1. (R Core Team), except for the probit regression and Pearson’s correlation which were performed in SPSS 27.0. All plots were drawn using the latter software as well.

Sample sizes were relatively small, data were often non-normally distributed, and between-group variance was often heteroscedastic, therefore robust statistical methods implemented in the R package WRS2 were employed (Mair and Wilcox 2020). Instead of mean or median, a robust measure of central tendency was used, i.e., either a modified one-step *M*-estimator based on Huber’s Psi (est parameter set to “mom”) or a 20% trimmed mean (see below for details).

The avoidance response was determined in four different ways. In the first approach, we assumed that avoidance of contaminated soil was successful if animals spent more than half of the recording time on uncontaminated soil. Therefore, the percentage of time on uncontaminated soil was tested against a fixed value of 50% for each concentration and the control. To do so a robust one-sample test was applied using the function *onesampb()*, the *M*-estimator of central tendency, and 10,000 bootstraps (to estimate the 95% confidence intervals).

In the second approach, data on the time spent on uncontaminated soil were used to estimate the median effective concentration of pyrethrin (*EC_50_*) for avoidance response. Data were first transformed by a formula adapted from ISO (2008): AR = ((t_i_-t_c_)/t)*100 (t_i_ – time on contaminated soil; t_c_ – time on uncontaminated soil; t – total time of observation), and then the probit regression was applied. Negative values were considered as 0% of avoidance.

In the third approach, the avoidance response was assessed by the number of visits to contaminated soil. A robust two-way ANOVA was applied using the number of visits to contaminated soil as a dependent variable, while concentration treatment (0, 2.5, 5.0, 7.5, 10.0) and number of animals (isolated, paired) were used as independent categorical variables whose interaction was tested as well. For two-way ANOVA the function *pbad2way()*, the *M*-estimator of central tendency, and 5,000 bootstraps were used. Next, post-hoc comparisons were performed to find at which concentrations the response differed from the control in isolated (4 tests) and paired (4 tests) animals, as well as to find at which concentration treatments the response of isolated and paired animals was mutually different (5 tests). For this, robust independent two-sample tests were applied using the function *pb2gen()*, the *M*-estimator of central tendency, and 10,000 bootstraps (to estimate the 95% confidence intervals). *P*-values were adjusted via the method of Benjamini and Hochberg (1995).

In the fourth approach to estimate avoidance response, the location of animals at the end of the 3-h recordings was used. The percentage of animals on the uncontaminated soil was calculated.

Behavioural variables on isopod activity (duration, path length, average speed) were analysed as described for the third approach to avoidance response estimation. First, a robust two-way ANOVA was performed with the specific behavioural variable as a dependent variable, while concentration treatment and number of animals were used as independent categorical variables whose interaction was also tested. Upon significant effects, the same post-hoc comparisons procedure as stated above was applied. Additionally, Pearson’s correlation between the duration of locomotor activity and path length was calculated.

Data on average speed at contaminated (chamber B) and uncontaminated (chamber A) soil involved two measurements per individual. To account for repeated measurements, a robust two-way between-within subjects ANOVA was applied. Separate models were fitted for isolated and paired animals. In both, average speed was used as a dependent variable, while concentration treatment and arena chamber (A – uncontaminated, B – contaminated) were used as independent categorical variables whose interaction was also tested. In this case the function *bwtrim()* and the 20% trimmed mean as an estimate of central tendency were used. Next, post-hoc comparisons were performed to find at which concentration treatments average speed differed between the two arena chambers in isolated (5 tests) and paired (5 tests) animals. For this, robust dependent two-sample tests were applied using the function *yuend()* and the 20% trimmed mean as an estimate of central tendency. *P*-values were adjusted according to Benjamini and Hochberg (1995). Additionally, effect sizes, i.e., magnitude of between group mean differences, were calculated as proposed by Wilcox and Tian (2011).

## Results

### Avoidance response

#### Time spent on uncontaminated soil

**Figure 2.**
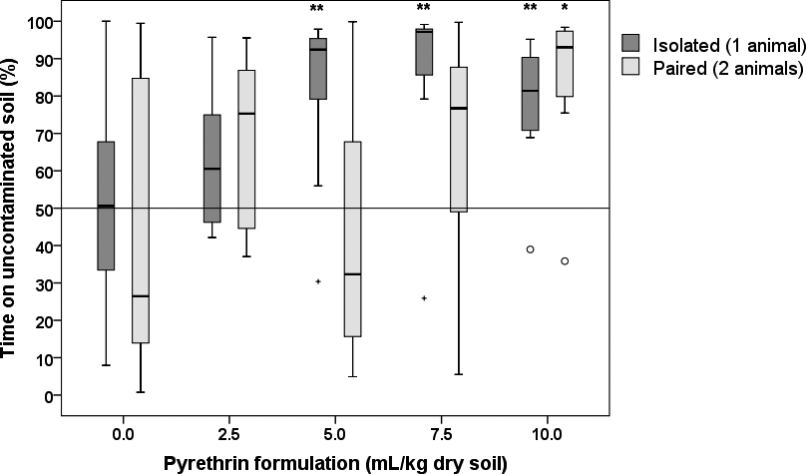
The percentage of time that *Porcellioscaber* spent on uncontaminated soil (chamber A) within the 3 h of observation. In a free-choice experiment isolated or paired animal could select between soil contaminated with pyrethrin and uncontaminated soil. Key: box: 25^th^, 50^th^, and 75^th^ percentile; whiskers: value ≤ 1.5 IQR; o – outlier: 3 IQR ≤ value > 1.5 IQR; + – extreme: value > 3 IQR; * – significantly different than 50%, *p* < 0.05; ** – as previous, but *p* < 0.01.

Isolated animals of the control group spent, on average, the same amount of time in both chambers containing uncontaminated soil (Suppl. material 1: Table S1, Fig. 2). This was the case also in the control group of paired animals although some preference for chamber B was noticed.

Isolated animals showed avoidance behaviour to 5.0, 7.5, and 10.0 mL of pyrethrin formulation per kg dry soil as they spent significantly more time on uncontaminated soil (Suppl. material 1: Table S1, Fig. 2). In contrast, paired animals spent significantly more time on uncontaminated soil only at the highest concentration used, i.e., 10.0 mL/kg dry soil.

#### EC_50_

The estimated *EC_50_* for isolated animals was 2.8 mL of pyrethrin formulation per kg dry soil (95% confidence interval: 2.7–5.0 mL/kg) while *EC_50_* for paired animals was 7.9 mL of pyrethrin formulation per kg dry soil (95% CI: 5.1–21.4 mL/kg), much higher than for isolated animals.

#### Number of visits to contaminated soil

The ANOVA showed that the number of visits to contaminated soil differed significantly between concentration treatments (*p* = 0.01) and number of animals (*p* = 0.006). However, we found no interaction effect between these two variables (*p* = 0.189), meaning that the difference between isolated and paired animals did not differ among concentration treatments.

**Figure 3.**
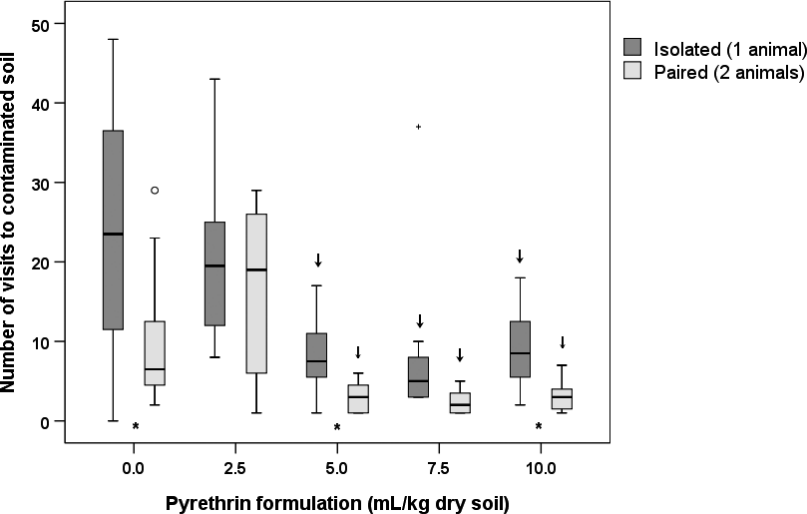
The number of visits to contaminated soil (chamber B) that *Porcellioscaber* made during the 3 h of observation. In a free-choice experiment, isolated or paired animals could select between soil contaminated with pyrethrin and uncontaminated soil. Key: box: 25^th^, 50^th^, and 75^th^ percentile; whiskers: value ≤ 1.5 IQR; o – outlier: 3 IQR ≤ value > 1.5 IQR; + – extreme: value > 3 IQR; ↓ – significantly lower than control, *p* < 0.05; * – significant difference between isolated and paired animals, *p* < 0.05; ** – as previous, but *p* < 0.01.

Post-hoc comparisons between different pyrethrin concentrations and the control revealed that in both isolated and paired animals the number of visits to contaminated soil significantly decreased at concentrations of 5.0 mL of pyrethrin formulation per kg dry soil or higher (Suppl. material 1: Table S2, Fig. 3). Moreover, post-hoc comparisons between isolated and paired animals at the same concentration treatment showed that paired animals visited contaminated soil less frequently compared to isolated animals in the control as well as at 5.0 and 10.0 mL/kg dry soil (Suppl. material 1: Table S2, Fig. 3). The same trend was observed also at 7.5 mL/kg dry soil, but the difference was only marginally statistically significant.

#### Location of animals after 3 hours

After 3 h, six isolated and five paired animals of control groups were found in chamber A, and the rest in chamber B. The location of pyrethrin exposed animals did not correspond to the concentrations of pyrethrin in soil (Table 1), or to the time spent on uncontaminated soil (Fig. 2). The highest avoidance in isolated animals was recorded at 5.0 mL/kg dry soil, while no avoidance was noticed at 10.0 mL/kg. In paired animals the highest avoidance was recorded at 7.5 mL/kg dry soil.

**Table 1.** The percentage of animals (*Porcellioscaber*) located on uncontaminated soil (chamber A) at the end of 3 h observation. In a free-choice experiment, animals could select between soil contaminated with pyrethrin and uncontaminated soil.

Pyrethrin concentration (mL/kg dry soil)	Isolated animals (%)	Paired animals (%)
0	50	42
2.5	50	62.5
5.0	92	25
7.5	75	87.5
10.0	50	75

### Activity of animals

#### Locomotor activity

##### Duration of locomotor activity

**Figure 4.**
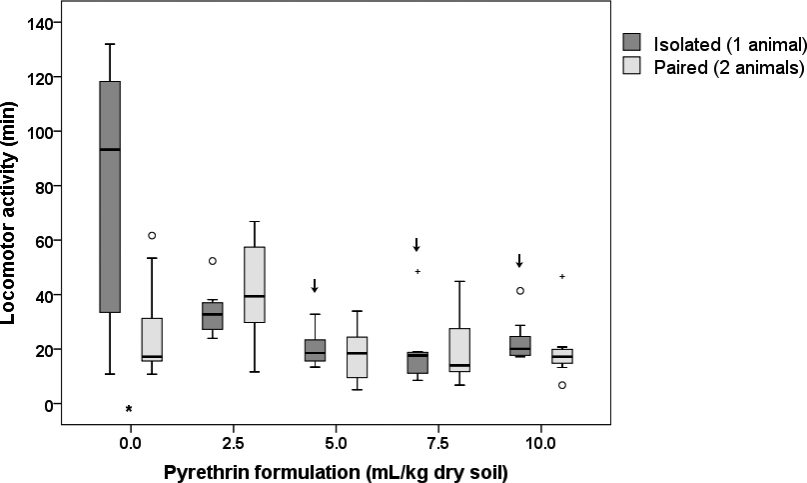
Duration of locomotor activity of *Porcellioscaber* within the 3 h of observation. In a free-choice experiment, isolated or paired animals could select between soil contaminated with pyrethrin and uncontaminated soil. Key: box: 25^th^, 50^th^, and 75^th^ percentile; whiskers: value ≤ 1.5 IQR; o – outlier: 3 IQR ≤ value > 1.5 IQR; + – extreme: value > 3 IQR; ↓ – significantly lower than control, *p* < 0.05; * – significant difference between isolated and paired animals, *p* < 0.05.

In general, the animals were locomotory active from several minutes up to 1 h (Fig. 4). An exception were the control isolated animals, where more than half of the animals were locomotory active more than 1 h, some animals even more than 2 h. The ANOVA showed that the duration of locomotor activity differed significantly between concentration treatments (*p* = 0.019) and the number of animals (*p* = 0.035), but we found no interaction effect between these two variables (*p* = 0.165). The latter meaning that the difference between isolated and paired animals did not differ among concentration treatments.

Post-hoc comparisons between different pyrethrin concentrations and the control revealed that in isolated animals, locomotor activity decreased when exposed to pyrethrin formulation in soil (Suppl. material 1: Table S3, Fig. 4). The trend was statistically significant at all concentrations but 2.5 mL/kg dry soil. This was not the case with paired animals, which exhibited similar locomotor activity in control and treatment groups, and were most locomotory active when exposed to 2.5 mL of pyrethrin formulation per kg dry soil. Moreover, post-hoc comparisons between isolated and paired animals at the same concentration treatment showed significantly higher locomotor activity than paired animals (Suppl. material 1: Table S3, Fig. 4). However, when exposed to pyrethrin, the locomotor activity duration did not differ between isolated and paired animals.

##### Path length

**Figure 5.**
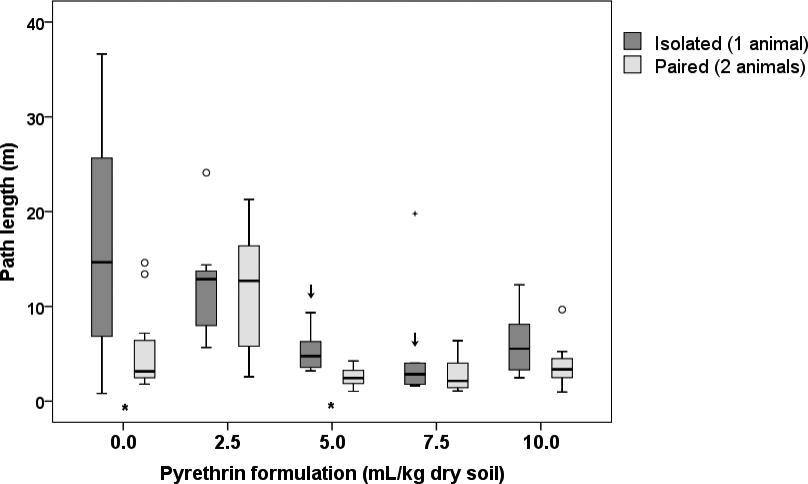
Overall length of the path that *Porcellioscaber* walked within the 3 h of observation. In a free-choice experiment, isolated or paired animals could select between soil contaminated with pyrethrin and uncontaminated soil. Key: box: 25^th^, 50^th^, and 75^th^ percentile; whiskers: value ≤ 1.5 IQR; o – outlier: 3 IQR ≤ value > 1.5 IQR; + – extreme: value > 3 IQR; ↓ – significantly lower than control, *p* < 0.05; * – significant difference between isolated and paired animals, *p* < 0.05.

The length of the path that the animals walked during the observation correlated with the duration of locomotor activity (Pearson correlation: *r* = 0.893, *p* < 0.001) and varied from 0.8 m up to nearly 37 m (Fig. 5). The ANOVA showed that the path length differed significantly between concentration treatments (*p* = 0.009) and number of animals (*p* = 0.01). However, no interaction effect was found between these two variables (*p* = 0.168), meaning that the difference between isolated and paired animals did not differ among concentration treatments.

Post-hoc comparisons between different pyrethrin concentrations and the control, revealed that in isolated animals the path length decreased with increasing concentration of pyrethrin formulation in soil, but was significantly lower than the control only at concentrations of 5.0 and 7.5 mL/kg dry soil (Suppl. material 1: Table S4, Fig. 5). In paired animals exposed to pyrethrin, the path length did not differ from the control at any concentration, but animals exposed to 2.5 mL/kg dry soil made the longest path. Furthermore, post-hoc comparisons between isolated and paired animals at the same concentration treatment showed no clear pattern in their mutual differences (Suppl. material 1: Table S4, Fig. 5). However, at the control and 5.0 mL/kg dry soil, the path length was significantly higher in isolated animals compared to paired animals.

##### Average speed

**Figure 6.**
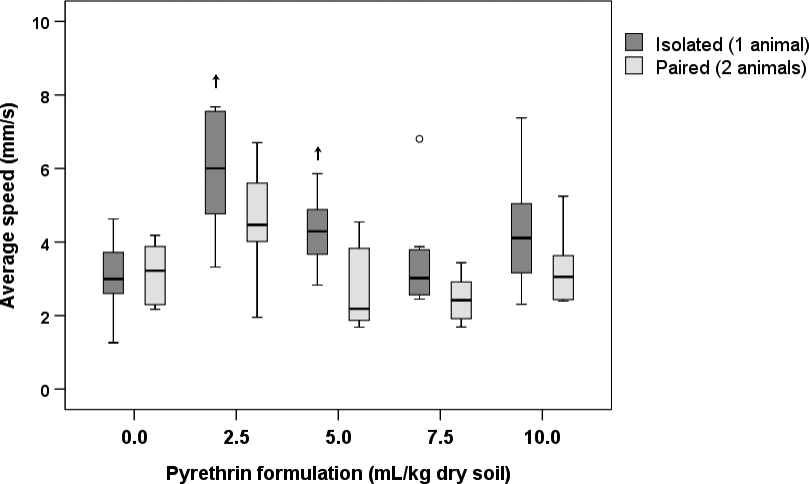
Average speed of locomotion of *Porcellioscaber* within the 3 h of observation. In a free-choice experiment, isolated or paired animals could select between soil contaminated with pyrethrin and uncontaminated soil. Key: box: 25^th^, 50^th^, and 75^th^ percentile; whiskers: value ≤ 1.5 IQR; o – outlier: 3 IQR ≤ value > 1.5 IQR; + – extreme: value > 3 IQR; ↑ – significantly higher than control, *p* < 0.05.

The average speed of locomotion of control animals was 1.3–4.6 mm/s in isolated animals and 2.2–4.2 mm/s in paired animals (Fig. 6). The ANOVA showed that average speed differed significantly between concentration treatments (*p* = 0.002) and number of animals (*p* = 0.003), but no interaction effect between these two variables was found (*p* = 0.238). The latter meaning that the difference between isolated and paired animals did not differ among concentration treatments.

Post-hoc comparisons between different pyrethrin concentrations and the control revealed that in isolated animals the average speed significantly increased at 2.5 and 5.0 ml/kg dry soil (Suppl. material 1: Table S5, Fig. 6). In pyrethrin exposed paired animals, the average speed was not significantly different from the control at any concentration. In addition, post-hoc comparisons between isolated and paired animals at the same concentration treatment showed non-significant differences in average speed in all cases (Suppl. material 1: Table S5, Fig. 6). However, the general pattern of higher average speeds in isolated compared to paired animals exposed to pyrethrin was supported by the significant ANOVA result (see above).

**Figure 7.**
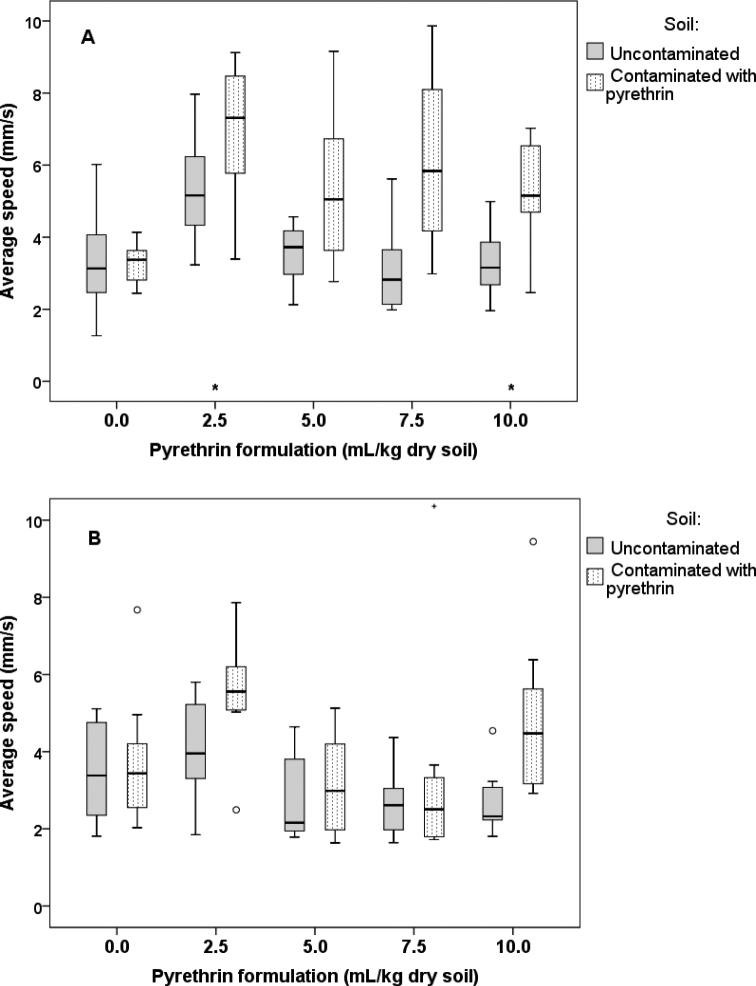
Average speed of locomotion of *Porcellioscaber* on uncontaminated soil (chamber A) and soil contaminated with pyrethrin (chamber B) for isolated animals (**A**) and paired animals (**B**). In a free-choice experiment, isolated or paired animals could select between soil contaminated with pyrethrin and uncontaminated soil. In plot A, two extreme values for chamber B are not shown: at conc. 2.5 (value = 12.99) and conc. 10 (value = 25.77). Key: box: 25^th^, 50^th^, and 75^th^ percentile; whiskers: value ≤ 1.5 IQR; o – outlier: 3 IQR ≤ value > 1.5 IQR; + – extreme: value > 3 IQR; * – significant difference between uncontaminated and contaminated soil, *p* < 0.05.

Further analyses focused on average speed of isolated and paired animals in the two arena chambers (uncontaminated vs. contaminated) at different pyrethrin concentrations. The ANOVA for isolated animals showed that their average speed depended on the arena chamber (*p* = 0.001) and concentration (*p* = 0.015), but the interaction of these two variables had no significant effect on the response (*p* = 0.268). The latter meaning that the difference in average speed between uncontaminated and contaminated soil did not differ among concentration treatments. Post-hoc comparisons revealed that isolated animals moved significantly faster on contaminated soil than on uncontaminated soil at 2.5 and 10.0 mL of pyrethrin formulation per kg dry soil (Suppl. material 1: Table S6, Fig. 7A). The same trend was observed at 5.0 and 7.5 mL/kg dry soil, but the differences were only marginally significant. However, note that the effect sizes were large also for the latter two comparisons.

The ANOVA for paired animals showed that their average speed depended on the arena chamber (*p* = 0.003) and concentration (*p* = 0.007), as well as the interaction of these two variables (*p* = 0.045). The significant interaction effect reveals that the difference in average speed between uncontaminated and contaminated soil differed among concentration treatments. Post-hoc comparisons revealed that paired animals did not move significantly faster on contaminated soil compared to uncontaminated soil at any concentration treatment, although marginally significant differences and large effect sizes in this direction were observed at concentrations 2.5 and 10.0 mL/kg dry soil (Suppl. material 1: Table S6, Fig. 7B). These are most likely also the reason for the significant interaction effect observed in ANOVA (see above).

#### Non-locomotor activity

**Figure 8.**
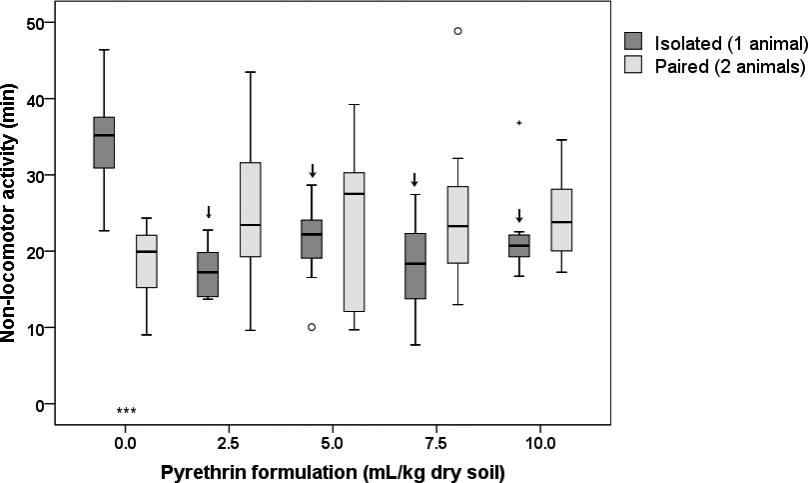
Duration of non-locomotor activity of *Porcellioscaber* within the 3 h of observation. In a free-choice experiment, isolated or paired animals could select between soil contaminated with pyrethrin and uncontaminated soil. Key: box: 25^th^, 50^th^, and 75^th^ percentile; whiskers: value ≤ 1.5 IQR; o – outlier: 3 IQR ≤ value > 1.5 IQR; + – extreme: value > 3 IQR; ↓ – significantly lower than control, *p* < 0.01; *** – significant difference between isolated and paired animals, *p* < 0.001.

Non-locomotor activity of animals lasted from 8 to 49 min (Fig. 8). The ANOVA showed that non-locomotor activity differed significantly between concentration treatments (*p* = 0.015), but not between the number of animals (*p* = 0.797). A significant interaction effect between these two variables (*p* < 0.001) was also observed, meaning that the difference between isolated and paired animals in non-locomotor activity differed among concentration treatments.

Post-hoc comparisons between different pyrethrin concentrations and the control revealed that in isolated animals, non-locomotor activity significantly decreased at all pyrethrin concentrations used (Suppl. material 1: Table S7, Fig. 8). This was not the case in paired animals for which no significant differences between the control and pyrethrin concentrations were observed. Post-hoc comparisons between isolated and paired animals at the same concentration treatment showed that in control the duration of non-locomotor activity was significantly higher in isolated animals (Suppl. material 1: Table S7, Fig. 8). When animals were exposed to soil contaminated with pyrethrin, non-locomotor activity tended to be higher in paired animals, but the differences were statistically non-significant at all concentrations. This pattern is most likely also the reason for the significant interaction effect observed in ANOVA (see above).

## Discussion

We investigated the influence of aggregation on avoidance behaviour and activity of *Porcellioscaber* exposed to contaminated soil. Individual animals (isolated) or animals in pairs were recorded for 3 h in two chambered arenas where they could select between uncontaminated soil and soil contaminated with the insecticide pyrethrin. Time spent on uncontaminated soil revealed more successful avoidance of contaminated soil in isolated than in paired animals. This measure of avoidance response was more sensitive and robust than the number of visits to contaminated soil or location of animals after a specific time since exposure. Animals unexposed to contaminated soil were significantly more active when isolated than when in pairs. This was evident from the duration of locomotory and non-locomotory activity. However, when exposed to pyrethrin the differences between isolated and paired animals decreased, although some differences in path length and average speed still indicated higher activity of isolated animals.

### Avoidance response

Although all animals started the experiment in chamber A, the control animals showed that the initial position of animals in the arena does not affect the time the control animals spent in each chamber of the arena (A or B, both containing uncontaminated soil). In published avoidance behaviour test protocols, animals were introduced into the test arenas differently: in the middle between control and test soils (Loureiro et al. 2005), randomly on both soils (Zidar et al. 2012; Škarkova et al. 2016) or on the uncontaminated soil (Zidar et al. 2019; the present study). Our result confirmed previous reports by Anselme (2013, 2015), that *P.scaber* shows high exploratory activity in a new environment and can explore both chambers of the arena in a short period of time.

During 3 h of exposure isolated isopods clearly avoided soil with pyrethrin formulation already at a concentration of 5.0 mL of formulation per kg dry soil, which roughly corresponds to 10.4 mg of pyrethrin per kg dry soil. This concentration is the lowest observed effective concentration (LOEC) in this study. According to our previous study (Zidar et al. 2012), this concentration can be considered as an upper sublethal concentration for *P.scaber*. In paired animals, the LOEC was twice as high, i.e., 20.8 mg/kg dry soil, at the concentration that is considered lethal to *P.scaber* (Zidar et al. 2012). Such weaker or delayed avoidance response in paired animals effectively means longer exposer to pyrethrin. Potentially, locomotor dysfunctions that the insecticide may induce makes the isopods even harder to retreat from the contaminated soil. Such positively reinforced feedback loop due to insecticide poisoning was reported previously for springtails exposed to dimethoate (Pereira et al. 2013).

The *EC_50_* value obtained in this study for isolated animals (2.8 mL of formulation or 5.9 mg of pyrethrin per kg dry soil) was almost twice lower than the *EC_50_* value obtained in the 48-h avoidance test with animals in groups (9.7 mg of pyrethrin per kg dry soil; Zidar et al. 2012). This was so even though in the latter study pyrethrin formulation contained piperonyl butoxide that enhances the effects of pyrethrin (Kakko et al. 2000). In paired animals the obtained *EC_50_* (7.9 mL of formulation or 16.5 mg of pyrethrin per kg dry soil) was almost 3× higher compared to isolated animals and also considerably higher than in our previous study (Zidar et al. 2012).

The frequency at which isolated animals visited contaminated soil decreased with the increased concentration of pyrethrin in soil and was in concordance with the time that animals spent on the contaminated soil. In paired animals the frequency of visits to contaminated soil was generally lower than that of isolated animals but their retention time on contaminated soil was much higher. As reported by Zidar et al. (2003), the number of visits does not necessarily reflect the exposure time.

The location of animals (on uncontaminated vs. contaminated soil) at a given time also does not necessarily reflect the avoidance response as has been reported previously (Odendaal and Reinecke 1997; Pereira et al. 2013). In our study, animals have often passed between chambers and after 3 h many animals were observed on contaminated soil, although they spent significantly more time on uncontaminated soil. However, the discrepancy between the animals’ location at a given time and avoidance success can be avoided by using average time spent on uncontaminated soil as a measure of avoidance (this study), or to record the location of animals during the exposure more frequently (see Madžarić et al. 2018).

Our results show that the accepted standard toxicity tests relying on avoidance behaviour of a group of individuals as an endpoint should probably be reconsidered when performed with gregarious animals like isopods that exhibit strong aggregation behaviour. These tests tend to underestimate the effect of the toxicant. The reason for this is twofold and originates from the dynamic hierarchy of the two independent stimuli of an opposite sign provided by the toxicant (negative) and the presence of conspecifics (positive) against the background environment (neutral). When isopods are introduced to a novel environment such as the test arena, they first explore it in approximately random movement. Note that the location of the negative stimuli is fixed while the positive stimuli move(s) randomly within the arena. When both stimuli appear on different halves of the arena the choice is clear. The dilemma arises when both negative and positive stimuli occur at the same arena half. In such scenarios, the animals’ response will depend on the relative strength of both stimuli. When toxicant concentration is high, it will prevail over conspecific attraction and animals will eventually aggregate at the optimal arena half. However, when toxicant concentration is low, conspecific attraction prevails over the negative effects of toxicant and animals will aggregate at the non-optimal arena half. The observed result is a lack of avoidance response interpreted as no effect of toxicant. However, when individual animals, and not groups, are tested at the same low toxicant concentration, they show clear avoidance signalling harmful effects. Thus, aggregation can mask the real effect of the toxicant and tests with groups of animals will tend to overestimate the effective toxicant concentration.

The real concern is that in natural populations animals will practically always be in a group. Groups of aggregating animals avoid high toxicant concentrations but are much less effective at avoiding low and moderate concentrations although harmful. Consequently, in a heterogeneously polluted environment these concentrations might eventually cause more damage as animals will be exposed to them longer and accumulate their negative effects, while they will retreat from higher concentrations. The standard toxicity tests with avoidance behaviour will however fail to reveal this. Thus, for gregarious animals we should rather estimate the effective concentration for both individual animals and those in a group. Although counterintuitive at first sight, the range of concentrations between these two values may be effectively most harmful to the natural populations.

Finally, group size and social composition of its members are additional factors that for sure add to the variation of toxicity tests results and should be considered in model species for which this is relevant and possible. Further investigation is needed in this direction. From the broadest perspective, aggregation of gregarious animals will likely affect the outcome and interpretation of any kind of choice tests with any kind of aversive (e.g., light, predator pheromones) or favourable (e.g., humidity, thigmotactic shelters) stimuli.

### Activity of animals

Locomotor behaviour of terrestrial isopods was recognised as a sensitive biomarker of exposure to different pollutants (Bayley et al. 1995, 1997; Sørensen et al. 1997; Engenheiro et al. 2005). Bayley et al. (1995) reported increased locomotory time, speed, and path length of *P.scaber* when exposed to sublethal concentrations of insecticide dimethoate. Later, studies showed that increased locomotor activity strongly correlate with the assimilated dimethoate and consequently acetylcholinesterase inhibition (Bayley and Beatrupe 1996; Jensen et al. 1997). On the other hand, Engenheiro et al. (2005) reported a decreased locomotory activity and path length of *Porcellionidespruinosus* exposed to higher concentrations of dimethoate. Decreased locomotor activity was recorded also in *Oniscusasellus* collected at polluted sites (Bayley et al. 1997; Sørensen et al. 1997). In our study, in contrast to the above examples, the choice for uncontaminated soil was offered, the locomotor and non-locomotor response and path length of isolated animals to pyrethrin contaminated soil was reduced. The activity of paired animals was generally low, in control and exposed animals, therefore exposed animals did not differ from the control animals. The total activity in control groups, non-locomotor and locomotor activity together, ranged 16–54 min/h in isolated and 7–27 min/h in paired animals. The activity of control isolated animals was comparable to control animals from the study of Bayley et al. (1995), while the activity of our paired animals was lower. The next obvious difference between isolated and paired isopods was the speed of locomotion on contaminated soil. Isolated animals were moving on contaminated soil much faster than on uncontaminated soil, but this was less obvious in paired animals. Faster locomotion in an environment with adverse stimuli is expected as it enhances the chance of finding a more suitable space, while slowing down can indicate the onset of favorable conditions (Fraenkel and Gunn 1961; Breed and Moore 2021). Although the rate of locomotion is often proportional to the intensity of the adverse stimulus, we did not observe such a response. On the other hand, different speed of locomotion on contaminated and uncontaminated soil also indicated that lower activity of exposed animals has not been caused by pyrethrin poisoning, as reported for dimethoate (Bayley and Beatrupe 1996).

## Conclusions

We conclude that:

- lower activity of aggregated animals leads to a less successful avoidance of moderately contaminated soil;
- aversive stimuli of pollution force animals to move faster on contaminated soil if not suppressed by aggregation behaviour;
- attraction between individuals might outweigh aversive stimuli of pollution leading to longer exposure to pollutant;
- aggregation behaviour should be accounted for when interpreting results of avoidance tests with groups of gregarious animals, which may underestimate the effect of pollutant.

## Supplementary materials

Supplementary material 1 Table S1–S7 Data type Statistical data Explanation note Avoidance behavior toxicity tests should account for animal gregariousness: a case study on isopod *Porcellioscaber*. https://binary.pensoft.net/file/685425This dataset is made available under the Open Database License (http://opendatacommons.org/licenses/odbl/1.0/). The Open Database License (ODbL) is a license agreement intended to allow users to freely share, modify, and use this Dataset while maintaining this same freedom for others, provided that the original source and author(s) are credited.Primož Zidar, Žiga Fišer
