# Supplementary material for: Avoidance behaviour toxicity tests should account for animal gregariousness: a case study on the terrestrial isopod Porcellioscaber
Source: Zookeys. 2022 May 18;1101:87–108. doi: 10.3897/zookeys.1101.76711 (PMC9848979; doi:10.3897/zookeys.1101.76711)
Supplement: Supplementary material 1 — Table S1–S7 [file zookeys-1101-087_article-76711__-s001.docx]

**Avoidance behavior toxicity tests should account for animal gregariousness: a case study on isopod *Porcellio scaber***

Primož Zidar^1^, Žiga Fišer^1^

^1^Department of Biology, Biotechnical Faculty, University of Ljubljana, Večna pot 111, SI-1000 Ljubljana, Slovenia

**SUPPLEMENTARY MATERIAL**

**Table S1:** Avoidance response as estimated by the percentage of time spent on uncontaminated soil: results of robust one-sample tests against a fixed value of 50%. Statistically significant *p*-values (*p* < 0.05) are bolded.

| **Concentration** | ***M*-estimator* [95% CI] ^†^** | ***p*-value** | ***p*-value adjusted^$^** |
| --- | --- | --- | --- |
|  |  |  |  |
| **SINGLE ANIMAL** |  |  |  |
| 0 | 52.6 [33.2, 68.1] | 0.952 | 0.952 |
| 2.5 | 62.6 [43.1, 75.9] | 0.321 | 0.402 |
| 5 | 91.2 [74.3, 96.0] | < 0.001 | **0.001** |
| 7.5 | 97.9 [85.7, 98.4] | 0.002 | **0.004** |
| 10 | 82.9 [64.5, 94.8] | 0.002 | **0.004** |
|  |  |  |  |
| **PAIRED ANIMALS** |  |  |  |
| 0 | 31.1 [10.1, 92.1] | 0.396 | 0.485 |
| 2.5 | 68.3 [40.7, 93.1] | 0.286 | 0.476 |
| 5 | 42.0 [8.1, 68.1] | 0.485 | 0.485 |
| 7.5 | 75.2 [45.8, 90.7] | 0.154 | 0.384 |
| 10 | 93.9 [68.9, 97.8] | 0.003 | **0.013** |

*Modifed one-step *M*-estimator based on Huber's Psi used as a robust measure of central tendency.

^†^95% confidence intervals are not adjusted and correspond to the unadjusted *p*-values.

^$^*P*-values were adjusted according to Benjamini and Hochberg (1995).

**Table S2:** Number of visits to contaminated soil (chamber B): results of post-hoc comparisons based on robust independent two-sample tests. Statistically significant *p*-values (*p* < 0.05) are bolded.

| **Comparison type** | ***M*-estimator* [95% CI] ^†^** | ***p*-value** | ***p*-value adjusted^$^** |
| --- | --- | --- | --- |
| Single animals: control vs. concentration |  |  |  |
| 1: 0 vs. 2.5 | 6.88 [-11.15, 19.41] | 0.532 | 0.576 |
| 1: 0 vs. 5 | 16.80 [3.05, 28.09] | 0.009 | **0.031** |
| 1: 0 vs. 7.5 | 19.17 [5.56, 31.40] | 0.011 | **0.031** |
| 1: 0 vs. 10 | 15.04 [1.38, 28.12] | 0.026 | **0.038** |
|  |  |  |  |
| Paired animals: control vs. concentration |  |  |  |
| 2: 0 vs. 2.5 | -9.9 [-21.27, 3.60] | 0.159 | 0.188 |
| 2: 0 vs. 5 | 3.6 [0.53, 11.96] | 0.023 | **0.038** |
| 2: 0 vs. 7.5 | 4.22 [1.50, 12.50] | 0.003 | **0.023** |
| 2: 0 vs. 10 | 3.47 [0.52, 11.75] | 0.026 | **0.038** |
|  |  |  |  |
| Within concentration: single vs. paired animals |  |  |  |
| 0: 1 vs.2 | 17.57 [3.34, 29.25] | 0.014 | **0.031** |
| 2.5: 1 vs.2 | 0.79 [-12.4, 17.75] | 0.863 | 0.863 |
| 5: 1 vs. 2 | 4.36 [1.83, 8.58] | 0.003 | **0.023** |
| 7.5: 1 vs. 2 | 2.62 [0.00, 5.50] | 0.055 | 0.072 |
| 10: 1 vs. 2 | 6.00 [1.29, 10.46] | 0.013 | **0.031** |

*A between-group difference in modified one-step *M*-estimators (based on Huber's Psi and used as a robust measure of central tendency).

^†^95% confidence intervals are not adjusted and correspond to the unadjusted *p*-values.

^$^*P*-values were adjusted according to Benjamini and Hochberg (1995).

**Table S3:** Duration of locomotor activity: results of post-hoc comparisons based on robust independent two-sample tests. Statistically significant *p*-values (*p* < 0.05) are bolded.

| **Comparison type** | ***M*-estimator* [95% CI] ^†^** | ***p*-value** | ***p*-value adjusted^$^** |
| --- | --- | --- | --- |
| Single animals: control vs. concentration |  |  |  |
| 1: 0 vs. 2.5 | 48.14 [-2.77, 92.77] | 0.079 | 0.172 |
| 1: 0 vs. 5 | 60.35 [11.00, 104.66] | 0.001 | **0.016** |
| 1: 0 vs. 7.5 | 64.45 [13.53, 108.64] | 0.002 | **0.016** |
| 1: 0 vs. 10 | 60.39 [9.83, 104.25] | 0.004 | **0.017** |
|  |  |  |  |
| Paired animals: control vs. concentration |  |  |  |
| 2: 0 vs. 2.5 | -22.15 [-42.17, -1.44] | 0.038 | 0.098 |
| 2: 0 vs. 5 | 1.33 [-8.74, 19.18] | 0.916 | 0.916 |
| 2: 0 vs. 7.5 | 6.98 [-13.84, 22.53] | 0.248 | 0.359 |
| 2: 0 vs. 10 | 2.01 [-5.90, 18.84] | 0.771 | 0.887 |
|  |  |  |  |
| Within concentration: single vs. paired animals |  |  |  |
| 0: 1 vs.2 | 59.95 [9.77, 105.91] | 0.010 | **0.032** |
| 2.5: 1 vs.2 | -10.33 [-28.63, 7.28] | 0.229 | 0.359 |
| 5: 1 vs. 2 | 0.94 [-7.18, 11.97] | 0.819 | 0.887 |
| 7.5: 1 vs. 2 | 2.48 [-21.30, 8.56] | 0.538 | 0.699 |
| 10: 1 vs. 2 | 1.58 [-6.43, 12.56] | 0.228 | 0.359 |

*A between-group difference in modified one-step *M*-estimators (based on Huber's Psi and used as a robust measure of central tendency).

^†^95% confidence intervals are not adjusted and correspond to the unadjusted *p*-values.

^$^*P*-values were adjusted according to Benjamini and Hochberg (1995).

**Table S4:** Path length: results of post-hoc comparisons based on robust independent two-sample tests. Statistically significant *p*-values (*p* < 0.05) are bolded.

| **Comparison type** | ***M*-estimator* [95% CI] ^†^** | ***p*-value** | ***p*-value adjusted^$^** |
| --- | --- | --- | --- |
| Single animals: control vs. concentration |  |  |  |
| 1: 0 vs. 2.5 | 5.1 [-6.50, 13.54] | 0.508 | 0.660 |
| 1: 0 vs. 5 | 10.63 [1.22, 20.41] | 0.015 | **0.049** |
| 1: 0 vs. 7.5 | 13.09 [2.94, 22.78] | 0.009 | **0.049** |
| 1: 0 vs. 10 | 9.71 [-0.03, 19.38] | 0.051 | 0.110 |
|  |  |  |  |
| Paired animals: control vs. concentration |  |  |  |
| 2: 0 vs. 2.5 | -8.67 [-14.20, -0.78] | 0.025 | 0.064 |
| 2: 0 vs. 5 | 0.48 [-0.67, 5.23] | 0.412 | 0.619 |
| 2: 0 vs. 7.5 | 1.19 [-1.5, 5.71] | 0.428 | 0.619 |
| 2: 0 vs. 10 | -0.06 [-2.71, 4.65] | 0.879 | 0.953 |
|  |  |  |  |
| Within concentration: single vs. paired animals |  |  |  |
| 0: 1 vs.2 | 12.76 [2.3, 22.04] | 0.013 | **0.049** |
| 2.5: 1 vs.2 | -1.01 [-7.21, 8.38] | 0.986 | 0.986 |
| 5: 1 vs. 2 | 2.61 [0.80, 3.96] | 0.003 | **0.036** |
| 7.5: 1 vs. 2 | 0.86 [-2.04, 2.29] | 0.654 | 0.773 |
| 10: 1 vs. 2 | 2.99 [-0.92, 5.45] | 0.185 | 0.344 |

*A between-group difference in modified one-step *M*-estimators (based on Huber's Psi and used as a robust measure of central tendency).

^†^95% confidence intervals are not adjusted and correspond to the unadjusted *p*-values.

^$^*P*-values were adjusted according to Benjamini and Hochberg (1995).

**Table S5:** Average speed: results of post-hoc comparisons based on robust independent two-sample tests. Statistically significant *p*-values (*p* < 0.05) are bolded.

| **Comparison type** | ***M*-estimator* [95% CI] ^†^** | ***p*-value** | ***p*-value adjusted^$^** |
| --- | --- | --- | --- |
| Single animals: control vs. concentration |  |  |  |
| 1: 0 vs. 2.5 | -2.89 [-4.88, -1.29] | 0.002 | **0.029** |
| 1: 0 vs. 5 | -1.24 [-2.07, -0.34] | 0.007 | **0.043** |
| 1: 0 vs. 7.5 | 0.04 [-1.44, 0.97] | 0.868 | 0.904 |
| 1: 0 vs. 10 | -0.78 [-2.45, 0.31] | 0.110 | 0.240 |
|  |  |  |  |
| Paired animals: control vs. concentration |  |  |  |
| 2: 0 vs. 2.5 | -1.84 [-3.05, -0.24] | 0.021 | 0.093 |
| 2: 0 vs. 5 | 0.93 [-1.09, 1.96] | 0.369 | 0.480 |
| 2: 0 vs. 7.5 | 0.69 [-0.37, 1.83] | 0.237 | 0.342 |
| 2: 0 vs. 10 | 0.19 [-1.13, 1.24] | 0.815 | 0.904 |
|  |  |  |  |
| Within concentration: single vs. paired animals |  |  |  |
| 0: 1 vs.2 | -0.07 [-1.15, 1.06] | 0.904 | 0.904 |
| 2.5: 1 vs.2 | 0.97 [-0.60, 3.41] | 0.127 | 0.240 |
| 5: 1 vs. 2 | 2.10 [0.13, 2.87] | 0.036 | 0.116 |
| 7.5: 1 vs. 2 | 0.58 [-0.27, 2.10] | 0.235 | 0.342 |
| 10: 1 vs. 2 | 0.90 [-0.44, 2.59] | 0.129 | 0.240 |

*A between-group difference in modified one-step *M*-estimators (based on Huber's Psi and used as a robust measure of central tendency).

^†^95% confidence intervals are not adjusted and correspond to the unadjusted *p*-values.

^$^*P*-values were adjusted according to Benjamini and Hochberg (1995).

**Table S6:** Average speed on uncontaminated and contaminated soil: results of post-hoc comparisons based on robust dependent two-sample tests. Statistically significant *p*-values (*p* < 0.05) are bolded.

| **Concentration** | **trimmed mean* [95% CI] ^†^** | **effect size^‡^** | ***p*-value** | ***p*-value adjusted^$^** |
| --- | --- | --- | --- | --- |
|  |  |  |  |  |
| **SINGLE ANIMAL** |  |  |  |  |
| 0: zone A vs. zone B | 0.07 [-0.69, 0.83] | 0.06 | 0.834 | 0.834 |
| 2.5: zone A vs. zone B | -1.94 [-2.86, -1.02] | 0.72 | 0.003 | **0.014** |
| 5: zone A vs. zone B | -1.58 [-3.29, 0.12] | 0.61 | 0.064 | 0.081 |
| 7.5: zone A vs. zone B | -3.17 [-6.01, -0.32] | 0.85 | 0.035 | 0.059 |
| 10: zone A vs. zone B | -2.23 [-3.53, -0.93] | 0.70 | 0.007 | **0.017** |
|  |  |  |  |  |
| **PAIRED ANIMALS** |  |  |  |  |
| 0: zone A vs. zone B | 0.10 [-1.42, 1.63] | 0.05 | 0.879 | > 0.999 |
| 2.5: zone A vs. zone B | -1.46 [-2.39, -0.52] | 0.71 | 0.010 | 0.052 |
| 5: zone A vs. zone B | -0.41 [-1.67, 0.84] | 0.17 | 0.437 | 0.728 |
| 7.5: zone A vs. zone B | 0.00 [-0.87, 0.87] | 0.00 | > 0.999 | > 0.999 |
| 10: zone A vs. zone B | -1.88 [-3.35, -0.41] | 0.81 | 0.021 | 0.054 |

*A between-group difference in 20% trimmed means (used as a robust measure of central tendency).

^†^95% confidence intervals are not adjusted and correspond to the unadjusted *p*-values.

^‡^Explanatory measure of effect size as proposed by Wilcox and Tian (2011). Estimates lower that 0.1, 0.3, and 0.5 are considered as small, medium, and large effect sizes, respectively.

^$^*P*-values were adjusted according to Benjamini and Hochberg (1995).

**Table S7:** Duration of non-locomotor activity: results of post-hoc comparisons based on robust dependent two-sample tests. Statistically significant *p*-values (*p* < 0.05) are bolded.

| **Comparison type** | ***M*-estimator* [95% CI] ^†^** | ***p*-value** | ***p*-value adjusted^$^** |
| --- | --- | --- | --- |
| Single animals: control vs. concentration |  |  |  |
| 1: 0 vs. 2.5 | 18.86 [12.97, 23.06] | < 0.001 | **< 0.001** |
| 1: 0 vs. 5 | 13.60 [8.30, 17.55] | < 0.001 | **< 0.001** |
| 1: 0 vs. 7.5 | 18.19 [10.70, 23.14] | < 0.001 | **< 0.001** |
| 1: 0 vs. 10 | 16.06 [9.74, 18.67] | 0.001 | **0.004** |
|  |  |  |  |
| Paired animals: control vs. concentration |  |  |  |
| 2: 0 vs. 2.5 | -5.65 [-14.07, 2.34] | 0.250 | 0.325 |
| 2: 0 vs. 5 | -4.02 [-13.85, 9.84] | 0.369 | 0.400 |
| 2: 0 vs. 7.5 | -2.34 [-13.64, 2.75] | 0.363 | 0.400 |
| 2: 0 vs. 10 | -4.91 [-10.64, 1.47] | 0.111 | 0.207 |
|  |  |  |  |
| Within concentration: single vs. paired animals |  |  |  |
| 0: 1 vs.2 | 16.63 [10.91, 20.62] | < 0.001 | **< 0.001** |
| 2.5: 1 vs.2 | -7.88 [-16.66, 0.15] | 0.055 | 0.118 |
| 5: 1 vs. 2 | -0.99 [-10.96, 12.17] | 0.621 | 0.621 |
| 7.5: 1 vs. 2 | -3.90 [-15.41, 2.93] | 0.240 | 0.325 |
| 10: 1 vs. 2 | -4.34 [-8.74, 2.77] | 0.171 | 0.277 |

*A between-group difference in modified one-step *M*-estimators (based on Huber's Psi and used as a robust measure of central tendency).

^†^95% confidence intervals are not adjusted and correspond to the unadjusted *p*-values.

^$^*P*-values were adjusted according to Benjamini and Hochberg (1995).

**References**

Benjamini Y, Hochberg Y (1995) Controlling the false discovery rate: a practical and powerful approach to multiple testing. Journal of the Royal statistical society: series B (Methodological), 57(1), 289-300. https://doi.org/10.1111/j.2517-6161.1995.tb02031.x

Wilcox RR, Tian TS (2011) Measuring effect size: a robust heteroscedastic approach for two or more groups. Journal of Applied Statistics, 38(7), 1359-1368. https://doi.org/10.1080/02664763.2010.498507
